# The advantages of domestic ear molding system in molding bilateral neonatal congenital auricular deformities

**DOI:** 10.1186/s12887-023-03916-3

**Published:** 2023-03-03

**Authors:** Jie Li, Junlong Tan, Denghua Yang, Liyan Chen

**Affiliations:** Department of Otorhinolaryngology, Fu Xing Hospital, Capital Medical University, No. 20 Fuxingmenwai Street, Xicheng District, Beijing, 100038 China

**Keywords:** Neonatal, Auricular deformity, Molding, Nonsurgical, Domestic

## Abstract

**Background:**

There are different types of ear molding devices on the market. However, due to high cost, the wide application of the ear molding is hindered, especially for children with bilateral congenital auricular deformities (CAD). This study is designed to correct the bilateral CAD with the flexible use of Chinese domestic ear molding system.

**Methods:**

Newborns diagnosed with bilateral CAD were recruited in our hospital from September 2020 to October 2021. For each subject, one ear wore a set of domestic ear molding system, while the contralateral ear used only matching Retractor and Antihelix Former. Medical charts were reviewed to collect data on the types of CAD, the incidence of complications, the initiation and duration of treatment, as well as the satisfaction after treatment. Treatment outcomes were graded into three levels: excellent, good, and poor, according to the improvement of auricular morphology evaluated by both doctors and parents, respectively.

**Results:**

A total of 16 infants (32 ears) were treated with the Chinese domestic ear molding system, which contains 4 cases with Stahl’s ear (8 ears), 5 cases with Helical rim deformity (10 ears), 3 cases with Cup ear (6 ears), 4 cases with Lop ear (8 ears). All infants accomplished the correction completely. Both parents and doctors were satisfied with the outcomes. No obvious complication was observed.

**Conclusions:**

Ear molding is an effective nonsurgical treatment for CAD. Molding with Retractor and Antihelix Former is simple and effective. Domestic ear molding system can be flexibly used in correcting bilateral CAD. With this approach, infants with bilateral CAD will benefit more in the near future.

## Background

Congenital auricular anomalies can be classified into malformations and deformities. Malformations are usually characterized as undeveloped pinnae with missing or mishappen extra skin or cartilage. Deformities are characterized by an abnormal ear shape without deficiencies in skin or cartilage of the pinna. These anomalies may have a lasting psychosocial impact as a consequence of teasing during childhood. Generally, malformations require surgery while most deformities can be corrected via ear molding in early time of life.

There is no consensus on the exact incidence of congenital auricular deformities (CAD), which varies racially from 6 to 57% [1, 2]. A Canadian study followed infants with CAD whose parents refused ear molding therapy, and reported that 67% did not correct spontaneously [3]. Up to date, ear molding with the external device is the safest and most effective nonsurgical therapy for newborns with CAD. With favorable prognosis and less complications, the ear molding provides the opportunity for preventing the development of associated psychological distress, avoiding the trauma, and reducing the cost of later surgical otoplasty in the future [4].

**Fig. 1:**
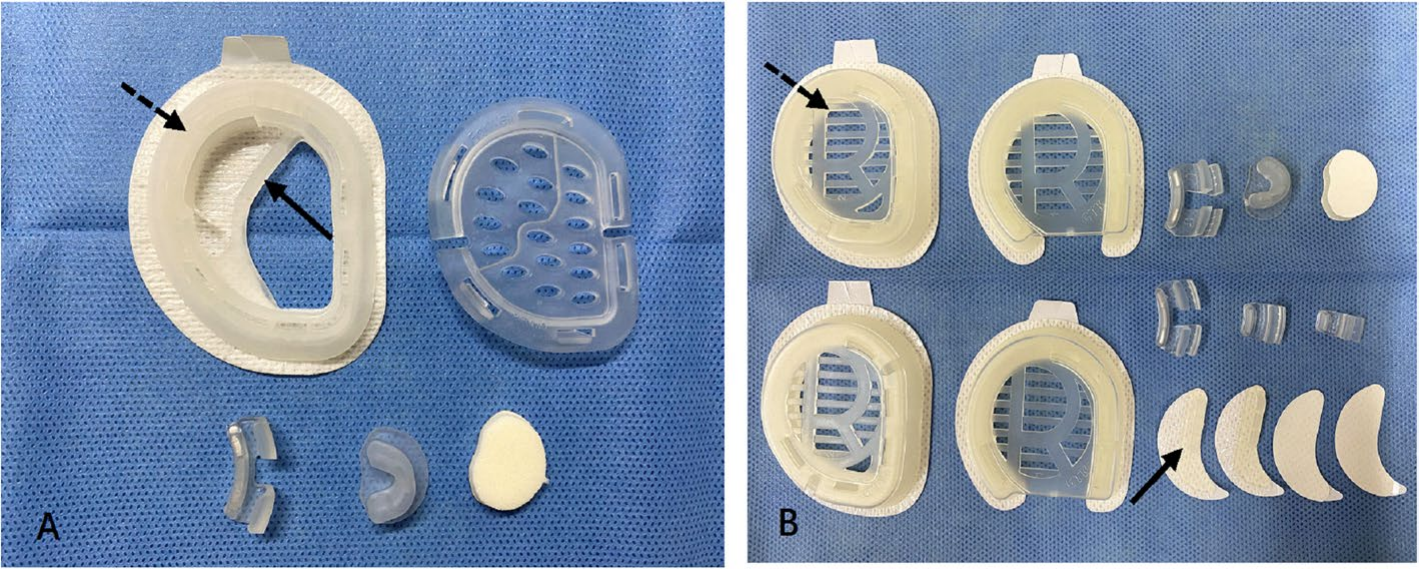
**A** The Antihelix Former (solid arrow) is connected with the Shell (dotted arrow) as shown in the EarWell Correction System; **B** The Antihelix Former (solid arrow) is separated from the Shell (dotted arrow) as shown in the GTK Ear Correction Kit

There are different types of ear molding devices on the market, mainly represented by EarWell correction system (Fig. 1A), which was first introduced to China in 2015. Recently, some domestic ear molding system for correcting CAD have been developed in China and are cheaper than the EarWell device. It is worth noting that, neither EarWell nor domestic ear molding system can be covered by any medical insurance in China. With the cost around 10,000 RMB for one EarWell device, the wide application of this ear molding is hindered. The price of the domestic ear molding system, which is about 2/3 the price of the EarWell device, is also not widely accepted, especially in rural or underdevelopment area in China. Mainly considering the economic reason, many parents would choose to wait for self-correction, especially for children with bilateral CAD, which may delay or even miss the correction opportunities.

This study is designed to correct the bilateral CAD with the flexible use of Chinese domestic ear molding system. For one ear, a full set of ear molding system was used to correct the auricular deformities, while for the contralateral ear, we used only the Retractor and Antihelix Former. By comparing the outcomes, we tried to evaluate the efficacy of domestic ear molding system and explore the feasibility of correcting bilateral CAD with one set of ear molding system, in order to decrease the cost of ear molding and benefit more infants.

## Methods

### Subjects

Newborns with bilateral CAD were recruited to the present study from September 2020 to October 2021 in our hospital. The inclusion criteria were as follows: (1) Newborns aged under 2 weeks who were diagnosed with bilateral CAD; (2) The periauricular skin was intact; (3) No improvement in auricle deformity was observed during 3 days after diagnose; (4) For economic or other reasons, the parents were willing to try correcting bilateral CAD with one set of domestic ear molding system; (5) All the newborns were full-term infants. Exclusion criteria: (1) Older than 2 weeks; (2) Congenital auricular malformation; (3) Poor compliance of parents who couldn’t regularly follow up; (4) Infants without passing the newborn hearing screening.

The study was approved by the hospital’s ethics committee. The informed consent was obtained from parents, specifically citing the possibility of local skin complications, failure to achieve the desired cosmetic result, and the possibility of need for future procedures. Clinical photographic documentation was obtained before, during and after treatment.

### Technique

A set of GTK Ear Correction Kit (Guangzhou T.K Medical Instrument Co., Ltd., China) was used in the present study. The correction kit includes four Shells, Four Antihelix Formers, two Retractors, two Conchal Formers, and some Adhesive Tape. (Fig. 1B) The most important difference and merit of the GTK Ear Correction Kit from the EarWell correction system is that the Antihelix Former of GTK Ear Correction Kit is separated from the Shell. There are four sizes of Antihelix Former in each Correction Kit, which can be trimmed as needed by doctors to the flexible application of the Antihelix Former on the helix of the ear. Newborns within the first 2 weeks of life often sleep during the correction procedure, and a simple swaddle is usually sufficient for immobilization. It is often helpful to advise parents to feed their infants shortly before the correction.

**Fig. 2:**
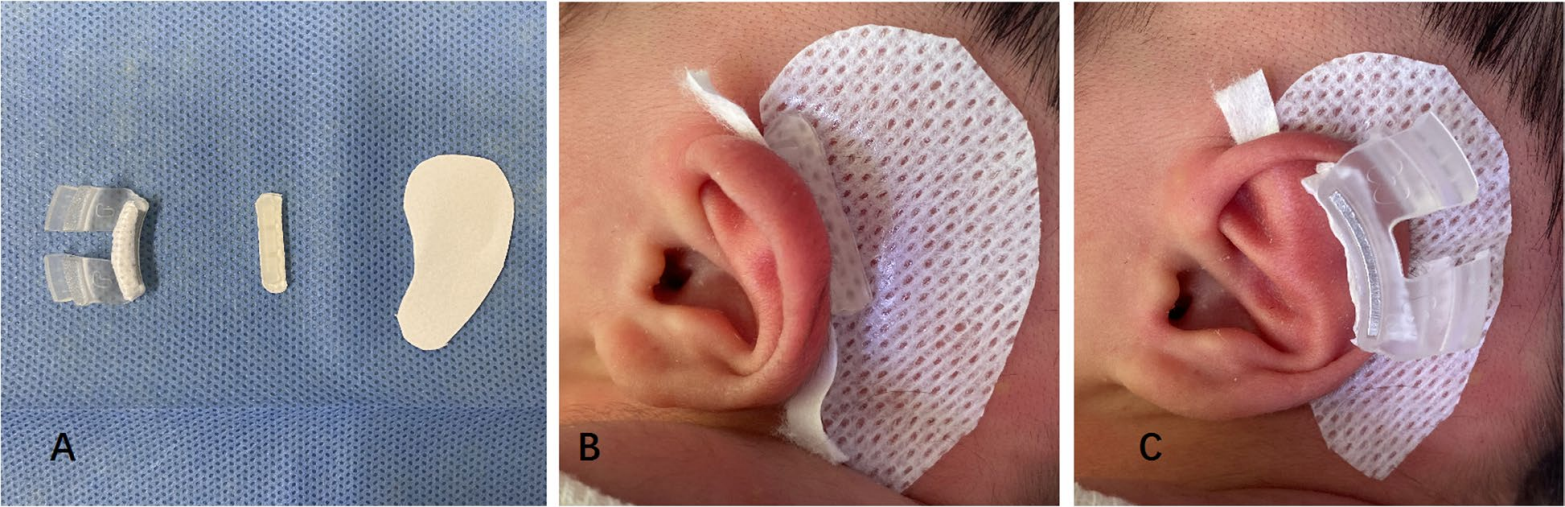
The Antihelix Former, Retractor and Double-Sided Adhesive Tape (**A**) can be all adjusted and then be fixed on the ear of the infants from **B** to **C**, step by step

Hair around the ear was shaved meticulously to allow for secure placement. The skin was then cleaned with 2 applications of rubbing alcohol. For one ear, an appropriate type of GTK Ear Correction Kit (medium size or large size) was customized to the patient’s auricle by bending and molding the material according to the characteristics of the deformities and secured in place. For the contralateral ear, the other Retractor from the same Correction Kit was applied to expand the helix and shape the scapha, while the other re-shaped Antihelix Former was equipped and maintained by Double-Sided Adhesive Tape (Fig. 2). After that, the accessories were covered with surgical tapes. What’s important, if any portion of the Retractor or Antihelix Former was in direct contact with skin, the area of anticipated contact area should be protected by double-sided adhesive tape first.

### Efficacy evaluation

The cosmetic outcomes were evaluated independently by both the parents and the doctor who participated in the molding process. It was graded to three levels after correction as follows:

- Excellent: the auricle shape was basically normal after correction, no appearance of original deformation.
- Good: 50–80% improvement in auricle shape, mild yet retention of original deformation.
- Poor: no or very little improvement in auricle shape, abnormal auricle shape with retention of original deformation.

### Follow-up

All infants wore the Correction Kit or its accessories 24 h a day and returned to the clinic for 7–10 days follow-ups until the auricle shape is normalized and stable, which usually took 3–5 weeks. The treatment was continued for another 2 weeks after the anomalies were corrected. Infants were followed up at 1 and 3 months after completion of treatment.

## Results

A total of 16 infants (32 ears) with bilateral CAD aged 3–10 days, were enrolled in this study, including 9 males and 7 females. Four types of CAD were included: Helical rim abnormality, Cup ear, Lop ear and Stahl’s ear. The average age for initiation of ear molding treatment was 4.37 ± 1.28 days，and mean therapy duration was 34.62 ± 6.03 days. All cases were successfully corrected, and the treatment duration was about 28–47 days. According to the improvement of auricular morphology, all infants were evaluated as excellent after treatment, with an effective rate of 100%. Moreover, no recurrence of auricular deformity was observed during next 3 months’ follow up in all cases.

### Lop ear

**Fig. 3:**
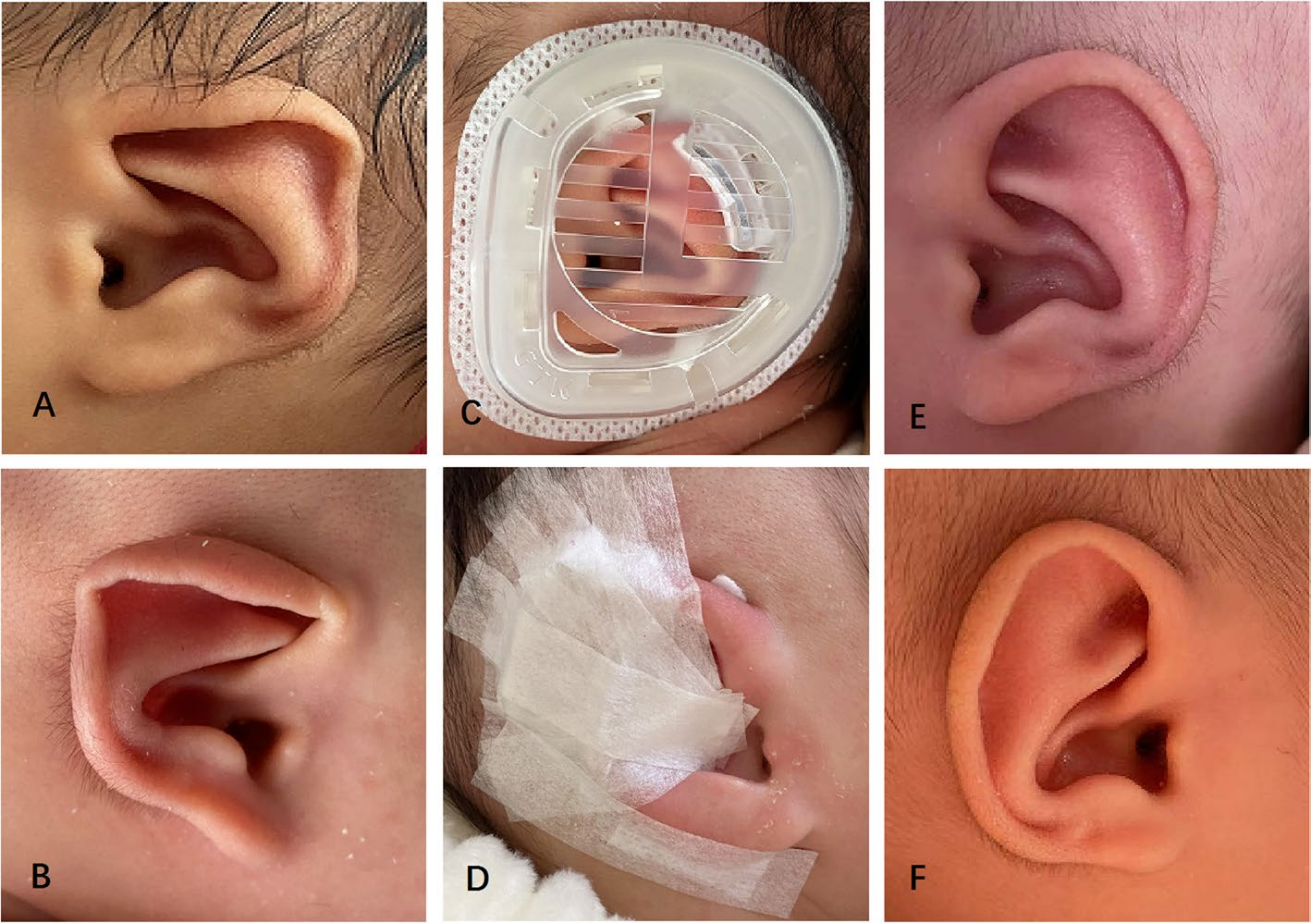
A 4-day-old boy with bilateral Lop ear: preoperative (**A** Left, **B** Right); correction in place (**C**); components in place (**D**); postoperative with favorable outcome after 37 days (**E** Left, **F** Right)

The lop ear is characterized that the upper part of the auricle is pendulous, which covers the upper foot of the antihelix. A total of 4 cases (8 ears) were treated with GTK Ear Correction Kit, and the results of both methods were excellent (Fig. 3).

### Helical rim deformity

**Fig. 4:**
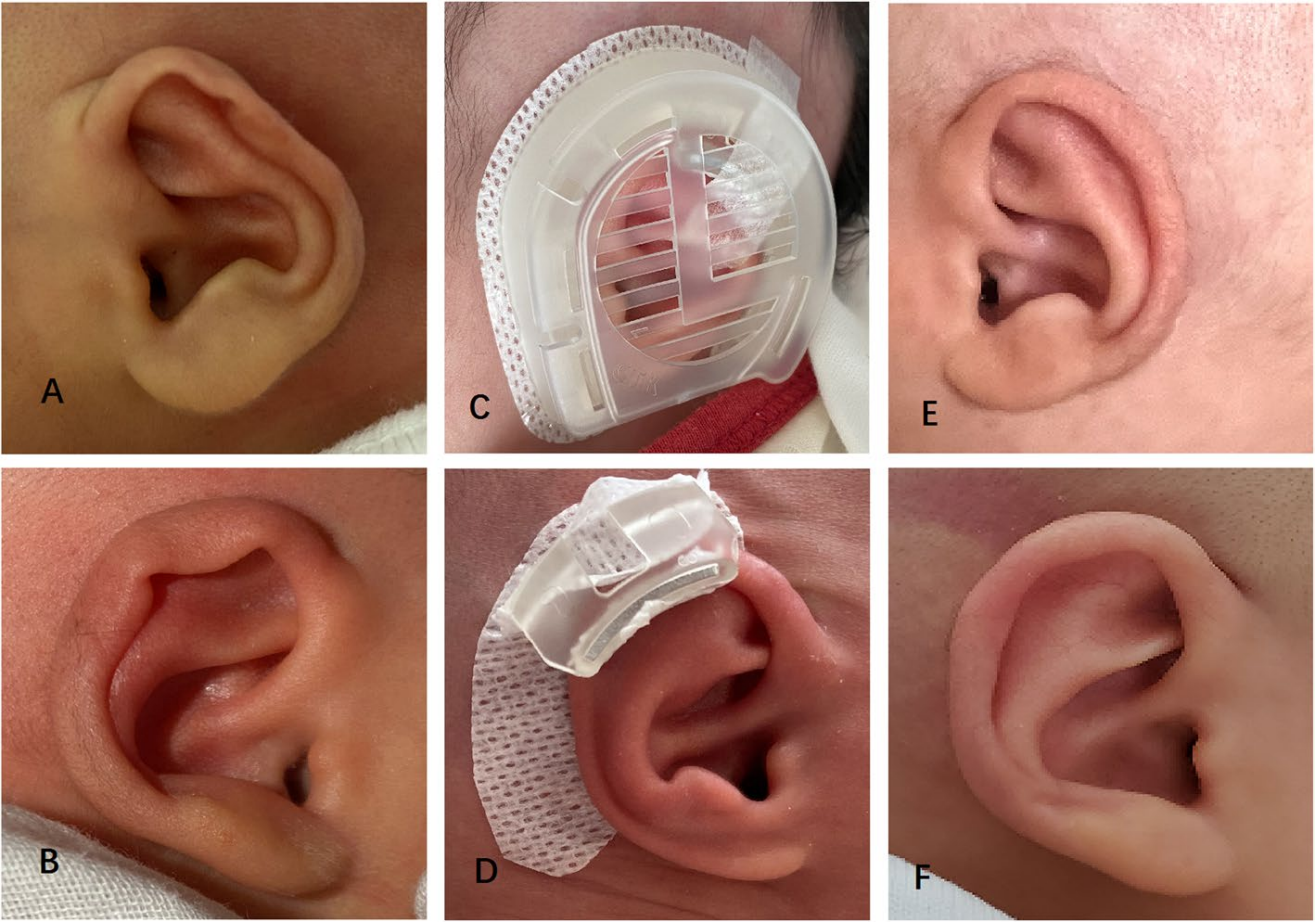
A 3-day-old boy with bilateral Helical Rim Deformity: preoperative (**A** Left, **B** Right); correction in place (**C**); components in place (**D**); postoperative with favorable outcome after 42 days (**E** Left, **F** Right)

The helical rim deformity is manifested by the poor curvature of the auricle, and the helix is flat or not present (Fig. 4). A total of 5 cases (10 ears) were molded in this study and the final outcomes were all excellent.

### Stahl 's ear

**Fig. 5:**
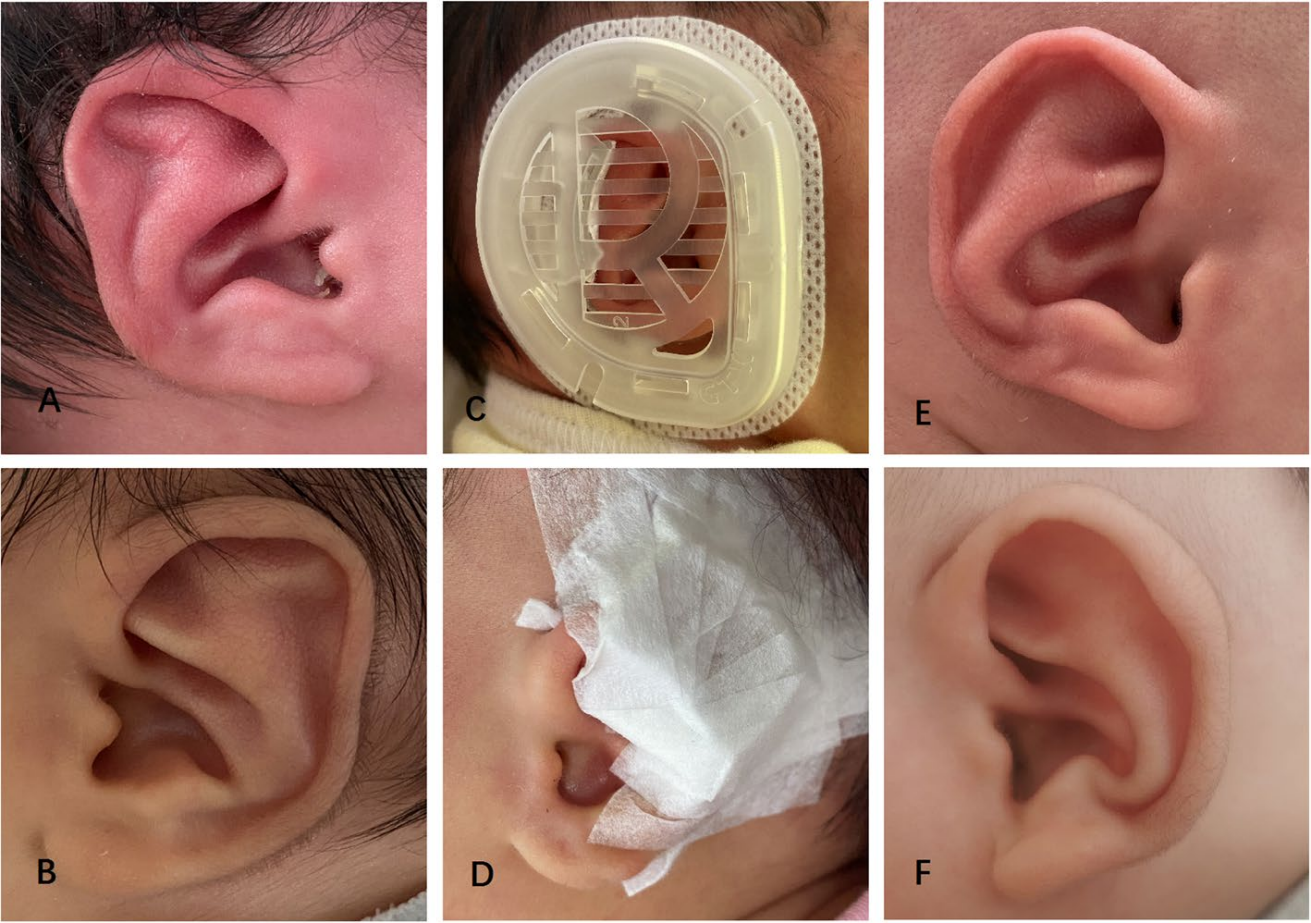
A 5-day-old boy with bilateral Stahl’s ear: preoperative (**A** Right, **B** Left); correction in place (**C**); components in place (**D**); postoperative with favorable outcome after 38 days (**E** Right, **F** Left)

Stahl’s ear is characterized by a prominent “3rd crus” causing disappearance of the scapha and flattening or abnormal bulge of the superior auricle. A total of 4 infants (8 ears) were treated with bilateral Stahl’s ear and the correction outcomes were all excellent (Fig. 5).

### Cup ear

**Fig. 6:**
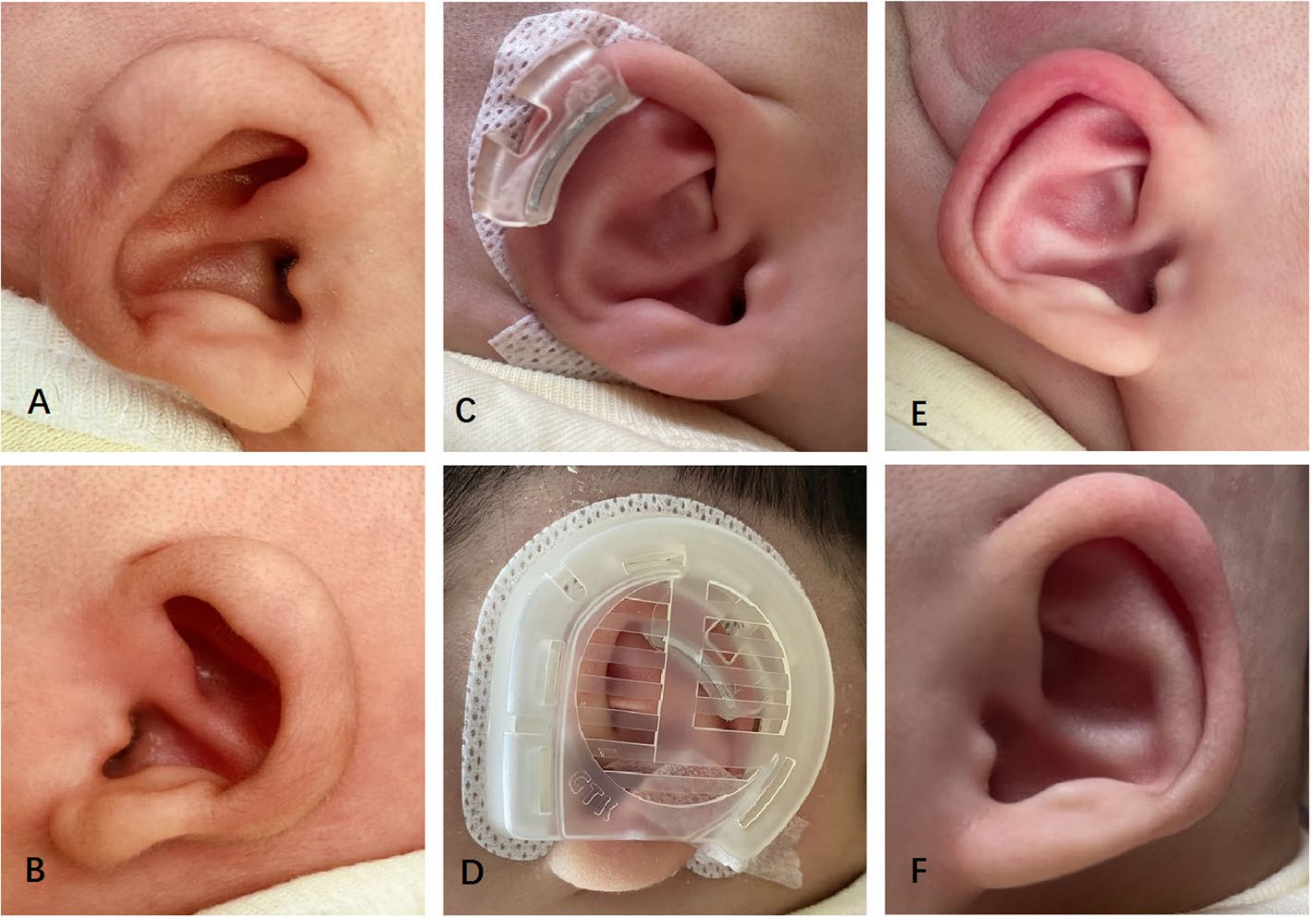
A 4-day-old girl with bilateral Cup ear: preoperative (**A** Right, **B** Left); components in place (**C**); correction in place (**D**); postoperative with favorable outcome after 47 days (**E** Right, **F** Left)

The cup ear is characterized by auricle leaning forward, which looked like a cup with an opening on the top when observed in the decubitus position. Meanwhile, the auricular rim is constricted, crimped and adherent, with the length of auricle becoming shorter. The triangular fossa becomes narrower but does not disappear. In this study, a total of 3 cases (6 ears) of cup ears were corrected with excellent outcome (Fig. 6).

### Complications

Periauricular skin complications were few and mild in all cases. In our study, most of infants only presented as periauricular redness and lesions immediately when the ear molding devices were removed caused by local traction and friction. Individually, we usually trained the parents to remove the correction 1 day in advance to heal the lesion, without delayed treatment. There were no complications such as eczema, pressure ulcers or exposed cartilage in our cases.

## Discussion

It is estimated that more than 5 million infants with CAD need to be corrected in China every year [5]. These deformities cause neither functional nor hearing issue, but result in psychological stress, self-abasement or social avoidance usually related to bullying [6]. Since the late 1980s, many authors demonstrated that permanent correction occurred by forcing the ear into the proper position for several weeks. As this treatment is initiated at an early age, children are no longer exposed to the psychosocial consequences of having an abnormal ear.

Up to date, the EarWell system and some Chinese domestic ear molding systems have shown excellent outcomes in correction of CAD. However, many infants in rural China, especially for those with bilateral CAD, fail to benefit from this nonsurgical treatment which is a high price and self-paying medical service.

During ear correction, it is found that the shape of the antihelix and scapha is very important to maintain the normal morphology of the auricle. Compared to EarWell or other ear molding system, the novelty of the GTK Ear Correction Kit is that the Antihelix Former is separated from the Shell and can be trimmed and adjusted to apply to different ear deformities. As far as bilateral CAD is concerned, one set of GTK Ear Correction Kit and its components can be used bilaterally instead of two sets of EarWell molding system should be used, reducing the cost obviously. Theoretically, the adjustable Retractor and Antihelix Former can counteract of the resistive forces of the abnormally inserted superior auricular muscle, such as shortened auricular transverse muscle and auricular oblique muscle [7]. Therefore, the skin is kept stretched and expanded. This is the main indicator of excellent results for ear molding in most studies. In our study, the Antihelix Former is used to revise the superior crus of the antihelix. The Retractor fixed in the scapha applies pressure to the helix, correcting a deformed rim helix and incurved scaphoid fossa. Therefore, this is useful for anomalies of the helical rim, lop ear, Stahl’s ear, prominent ear, cup ear and even cryptotia, to deepen the scapha, creating a well-defined superior crus.

It is widely accepted that time of treatment initiation is the most important factor for a successful correction case [8, 9]. Currently, delayed molding is not a common practice, with Daniali and colleagues citing a 50% failure rate for molding initiated after 3 weeks of age [10]. As a result of high level of circulating maternal estrogen, which reaches its peek within 72 hours after birth and decrease rapidly over the coming 6 weeks, the malleability of the auricular cartilage is hypothesized to be influenced during the neonatal period [11–13]. Early detection and diagnosis of CAD is crucial since ear molding takes advantage of the plasticity of auricular cartilage during the early time after birth.

Clinically, when a newborn was diagnosed with CAD who may be well response to the nonsurgical molding treatment, the practice is that the doctors and parents will conduct an observation period of 48 h–72 h to see what is going on with the CAD [14]. If there is no significant improvement in auricle morphology, ear molding will be advised. In this study, all the enrolled infants were younger at the initiation of correction. It can be noticed from the results that the treatment duration was short and the effective rate was as high as 100% when the initiation is within the first 2 weeks after birth. Moreover, it is recommended that the collaboration between midwifes, obstetricians, and pediatricians is key for early identification [15].

Previous studies have described rare complications in patients treated with ear molding system, including skin ulcers, dermatitis, infection, and bleeding, but no cartilage damage. The skin of the cranioauricular junction area or the helical rim would be worn by cradle or rigid retractors reported in other studies [16]. If no proper intervention, infection would follow behind. Usually, the cradle or shell can loosen with moisture. During our correction, thin cotton sheet is applied along the cranioauricular junction area to prevent moisture build up over time. Meanwhile, some double-sided adhesive tapes were applied to fix the appliance and protect skin. In our study, the most common complications were skin redness and rashes, which can recover spontaneously without any specific treatment. The lower incidence of complications might be related to early initiation and those medical skills to protect skin.

The notable shortcoming of using only appliances is that all molding takes place on the lateral surface of the ear. For Concha Crus ear, in addition to the Retractor and Antihelix Former, the Conchal Former should also be used for shaping. The Conchal Former reverses a protruding conchal strut, and the cap stabilizes the device, exerting consistent pressure on the retractor. In the future, related studies will be further designed to provide clinical data.

The major limitation of this study is the limited number of cases conducted. While ear molding has exhibited a high efficacy, it has yet to achieve widespread use due to a lack of recognition, delays in proper diagnosis, and an incorrect view that the ear deformities will self-correct over time. In addition to high cost, there have been some reasons for delays in receiving treatment by ear molding system, such as, unfamiliarity with the technique, which kinds of ear anomalies can be treated, and the suitable age for molding etc. Thus, to enhance widespread use in patients with CAD, several factors need to be considered: the cooperation between doctors in different departments, the extensive attention of the society and the cooperation of newborns’ parents.

To our limited knowledge, this study is the first one to explore the flexible use of Retractor and Antihelix Former in correcting bilateral CAD, which showed substantial efficacy. Our results reveal that only one set of domestic ear molding system can be used flexibly to correct bilateral CAD，reducing the cost for correction in bilateral cases. After clinical promotion, it is expected to benefit more infants. More and more infants with CAD will be included in such a research to establish the advantage of domestic ear molding system in bilateral ear correction in our future work.

## Conclusion

We confirm that the ear molding is a simple and effective procedure that should be offered more frequently to parents of affected neonates. The Chinese domestic ear molding system and its components had comparable therapeutic effects on neonatal CAD. Furthermore, it has advantage of molding bilateral CAD with lower cost.

## Data Availability

The datasets generated and/or analysed during the current study are available from the corresponding author on reasonable request.

## Abbreviation

CAD: Congenital auricular deformities
